# Lyme Disease: A Probably Underdiagnosed Cause of Mono-Arthritis

**DOI:** 10.5334/jbsr.2625

**Published:** 2021-11-22

**Authors:** Stijn Marcelis, Filip Vanhoenacker

**Affiliations:** 1 AZ Nikolaas UZA, BE; 2 AZ Sint-Maarten and University (Hospital) Antwerp/Ghent, BE

**Keywords:** Lyme disease, magnetic resonance imaging, arthritis

## Abstract

**Teaching point**: Meticulous anamnesis and a high index of suspicion is needed for the diagnosis of Lyme arthritis.

## Case presentation

**Figure 1:**
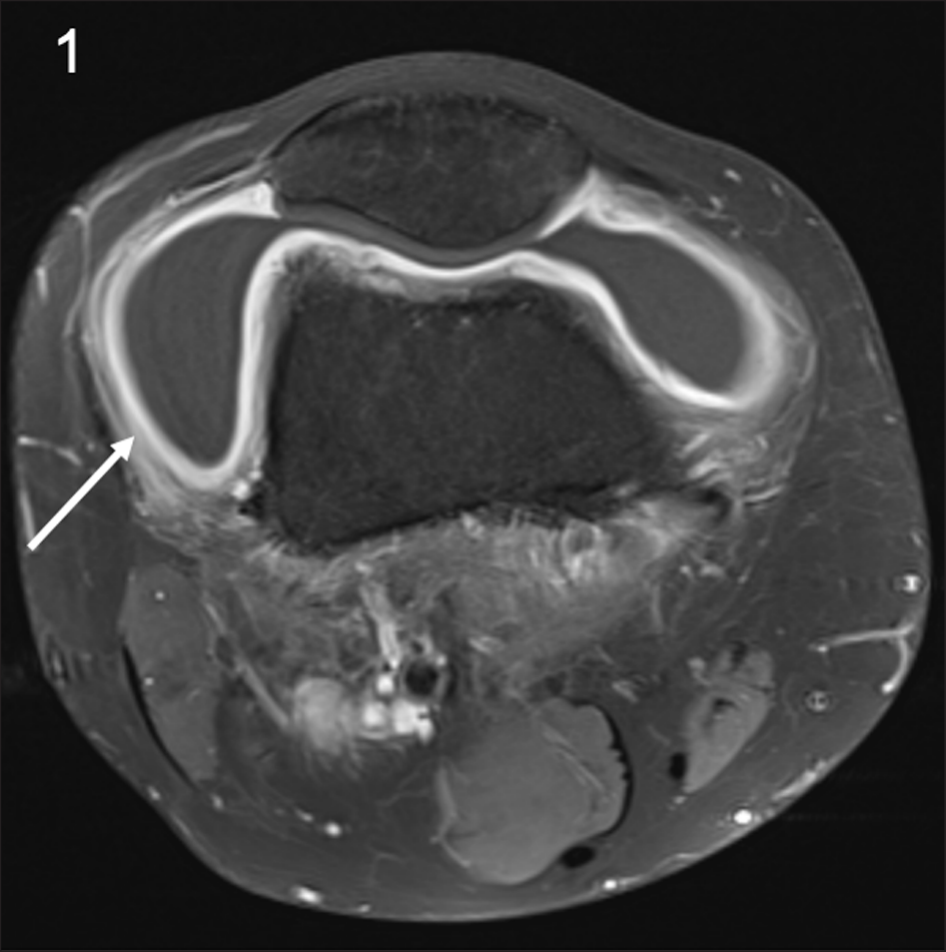


**Figure 2:**
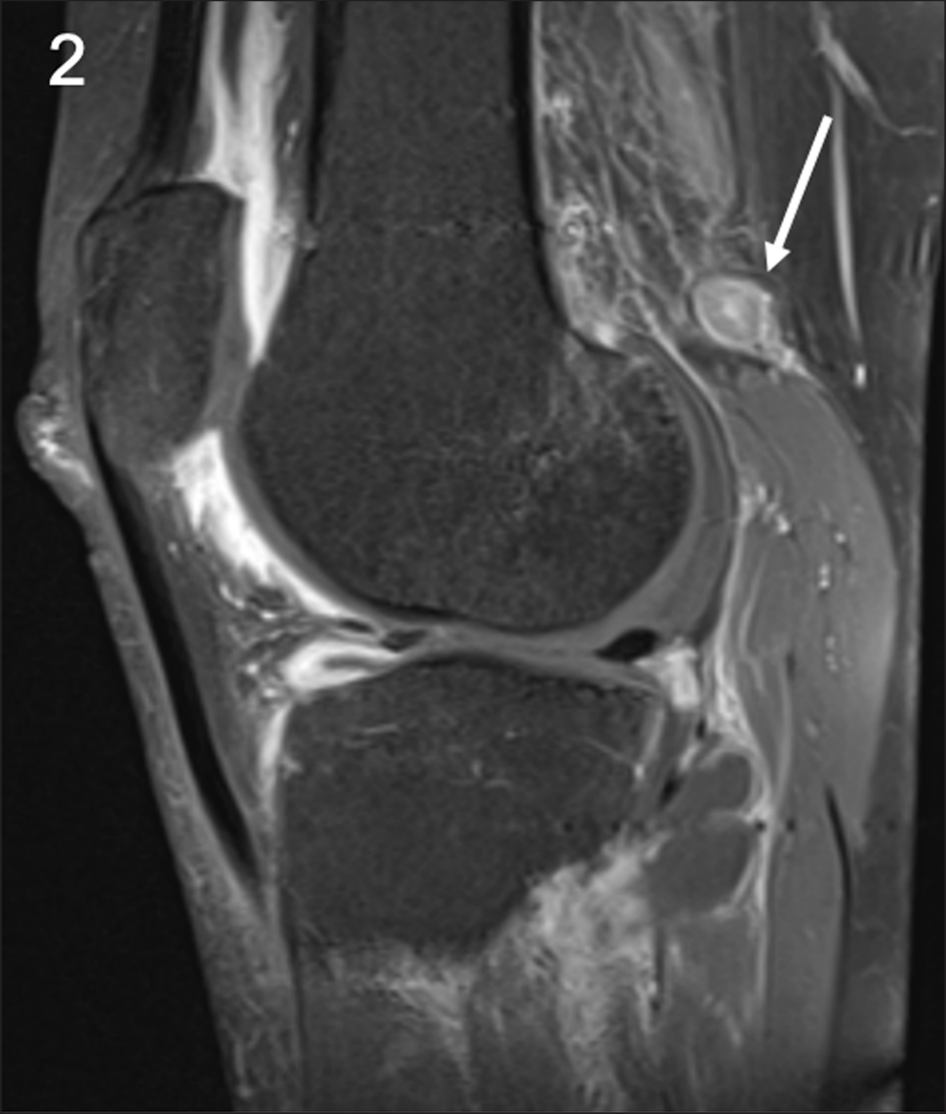


**Figure 3:**
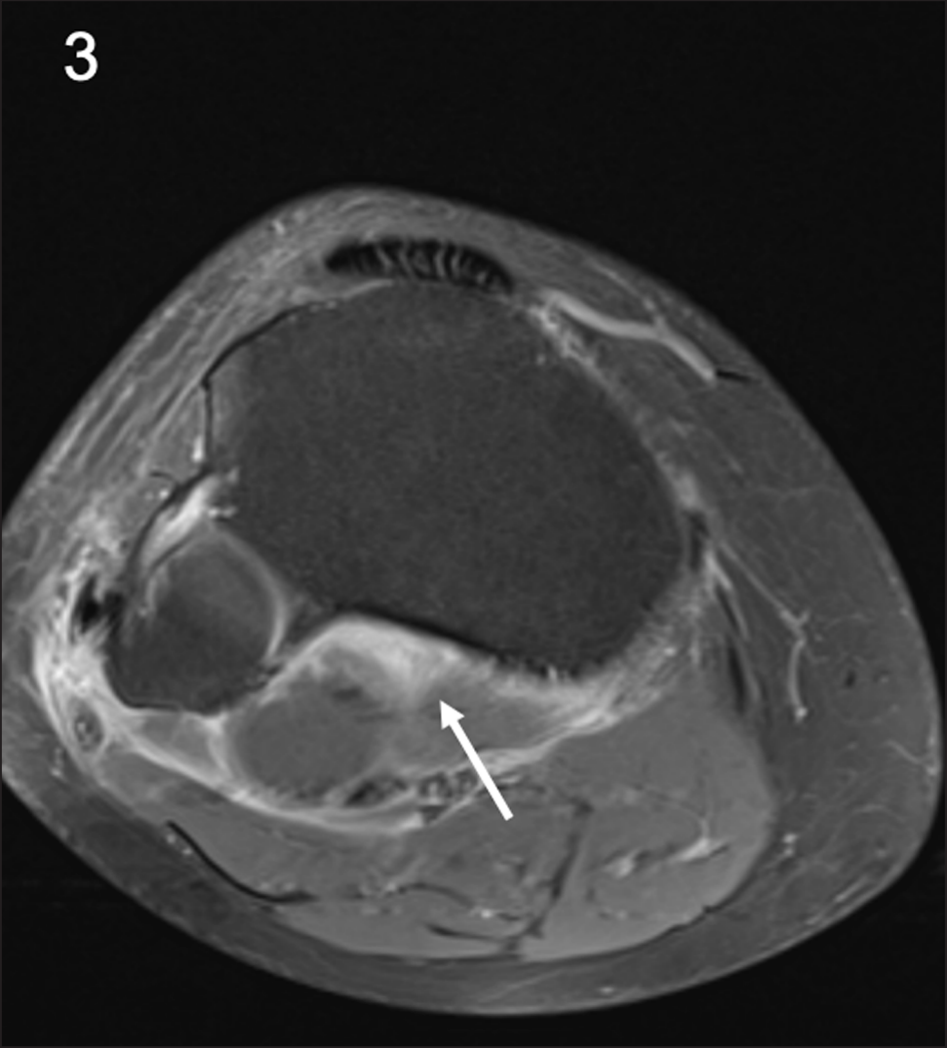


A 26-year-old man presented with acute knee pain for several days. He mentioned a similar period of knee pain last year since he started walking more intensively. Clinical examination revealed swelling of the knee. Routine magnetic resonance imaging (MRI) to exclude internal derangement revealed a large joint effusion. Follow-up MRI four months later showed a persistent joint effusion with diffuse enhancement and thickening of the synovium (***Figure 1***, arrow), enlarged lymph nodes in the popliteal fossa (***Figure 2***, arrow) and enhancement of the soleus muscle (***Figure 3***, arrow). Additional anamnesis revealed a history of serologically confirmed Lyme disease. The combination of synovitis, lymphadenopathy in the popliteal fossa, and serology led to the diagnosis of Lyme mono-arthritis.

## Comment

Lyme disease is a multisystemic disease caused by *Borrellia burgdorferi* and is the most common vector-borne illness transmitted by the bite of ticks. The estimated prevalence is around 100–130 per 100,000 in Europe and 20–100 per 100,000 in the United States. Around 80% of cases are reported between May and August. The highest incidence is seen in children between 5–9 years old [1].

The disease has three different stages with possible overlaps. Stage 1 (2–30 days) presents with flu-like symptoms and skin lesion. Stage 2 (1–4 months) consists of cardiac and neurologic symptoms. Stage 3 (several years) comes with arthritic and chronic neurologic symptoms.

Mono- and oligoarthritis is one of the most common manifestations, mostly affecting the knee, although the hip, ankle, elbow, and wrist may be affected [1]. Symptoms include fever, joint pain, and elevated acute phase reactants, similar to acute septic arthritis.

On MRI, Lyme arthritis, acute septic arthritis, and inflammatory arthritis may reveal joint effusion and synovial hypertrophy with hyperemia. There are three possible characteristics on MRI that could help to differentiate between Lyme or acute septic arthritis. The presence of lymphadenopathy in the popliteal fossa and myositis favor the diagnosis of Lyme arthritis. Subcutaneous edema on the other hand favors the diagnosis of septic arthritis; [1] however, it can be seen in patients with Lyme arthritis who underwent arthroscopy before imaging.

The final diagnosis of Lyme disease is based on specific serologic examination which may take up weeks. The treatment consists of an antibiotic course of four weeks.

Due to the similar clinical and laboratory presentation and radiological overlap between Lyme disease and acute septic arthritis, the diagnosis can be very challenging. Early differentiation should be made as there is an important therapeutic implication [1]. For this reason, we would like the emphasize the importance of a detailed anamnesis and clinical history to suggest the diagnosis in combination with imaging.
